# Bidirectional associations between mothers’ feeding practices and child eating behaviours

**DOI:** 10.1186/s12966-018-0644-x

**Published:** 2018-01-11

**Authors:** Elena Jansen, Kate E. Williams, Kimberley M. Mallan, Jan M. Nicholson, Lynne A. Daniels

**Affiliations:** 1 Centre for Children’s Health Research, 62 Graham Street (Level 6), South Brisbane, QLD 4101 Australia; 2 School of Early Childhood, Queensland University of Technology, Level 4 B Block, Kelvin Grove, QLD 4059 Australia; 3 School of Psychology, Australian Catholic University, 1100 Nudgee Road, Banyo, QLD 4014 Australia; 4 Judith Lumley Centre, La Trobe University, 215 Franklin Street, Melbourne, VIC 3000 Australia

**Keywords:** Feeding practices, Eating behaviour, BMI, Child, Parent, Longitudinal, Cross-lagged, Early childhood

## Abstract

**Background:**

This study examined bidirectional relationships between maternal feeding practices and child food responsiveness and satiety responsiveness from 2 to 5 years.

**Methods:**

Mothers (*N* = 207) reported their own feeding practices and child eating behaviours using validated questionnaires at child ages 2, 3.7, and 5 years. Cross-lagged analyses were conducted to test for bidirectional effects, adjusting for child BMI z-score (based on measured weight and height) at 14 months.

**Results:**

Eating behaviours and feeding practices showed strong continuity across the three time points. Maternal feeding practices (higher reward for behaviour [β = 0.12, *p* = 0.025] and lower covert restriction [β = −0.14, *p* = 0.008]) were prospectively associated with higher food responsiveness. Conversely, increased child satiety responsiveness was primarily prospectively associated with mothers’ feeding practices (increased structured meal timing [β = 0.11, *p* = 0.038], overt [β = 0.14, *p* = 0.010] and covert restriction [β = 0.11, *p* = 0.022]). The only exception was family meal setting, which was prospectively negatively associated with satiety responsiveness (β = −0.11, *p* = 0.035).

**Conclusion:**

While maternal feeding practices and child satiety and food responsiveness show strong continuity between child age 2 and 5 years, maternal feeding practices appear to be associated with child food responsiveness over time. Conversely, child satiety responsiveness, but not food responsiveness, may also be associated with maternal feeding practices over time. These results are consistent with interventions that provide feeding advice to parents on how to respond appropriately to individual child eating behaviour phenotype.

**Trial registration:**

ACTRN12608000056392. Registered 29 January 2008.

**Electronic supplementary material:**

The online version of this article (10.1186/s12966-018-0644-x) contains supplementary material, which is available to authorized users.

## Background

In response to the established need to prevent child obesity [1] research has considered a range of modifiable determinants of children’s eating behaviours, dietary intake and physical activity/inactivity that set the foundation for lifelong healthy or unhealthy habits. Parental feeding practices have been the focus of a number of recent child obesity prevention interventions [2] due to established links with child eating behaviours and weight status [3]. There is evidence that parents commonly use feeding practices that encourage children to eat for reasons other than hunger (e.g. reward for eating or other desired behaviours or regulate emotional state) or otherwise attempt to over-ride children’s hunger and satiety cues (e.g. pressure to eat) [4, 5]. These types of practices have been termed ‘coercive control’ [6] or ‘non-responsive’ [7, 8] feeding and may contribute to the development of childhood obesity by fostering obesogenic eating behaviours such as overeating [3, 9]. An alternative, more positive approach to feeding has been conceptualised as ‘authoritative’ feeding [7, 10] and combines two related operational components of parental feeding practice. Firstly, parents recognise and are appropriately responsive to child signals of huger and satiety (responsive feeding), and secondly they provide structure (routines and supportive limits, reduced distraction, predictable schedule) [6] within the feeding context that support both parent and child to attend to, communicate and then respond to child cues of hunger and satiety [5, 11–17]. The Trust model [13] theorises that the two components of authoritative feeding support and maintain the child’s capacity to self-regulate energy intake and develop healthy eating habits which in turn may be associated with reduction in child obesity risk.

Two child eating behaviours that have become of particular interest in this context are satiety responsiveness (i.e. fullness sensitivity reflecting good self-regulation in eating capability) and food responsiveness (i.e. tendency to overeat reflecting poor self- regulation in eating capability) [18]. Together low satiety responsiveness and high food responsiveness have been purported to be indicative of a ‘big appetite’, and both have been associated with overeating and overweight [19–21]. Recent evidence from twin studies has shown that these eating behaviours, which are commonly measured by the parent-report Children’s Eating Behaviour Questionnaire (CEBQ) [22], are highly heritable (genetic component between 63 and 89%) [19, 23, 24] and are stable across time points [25, 26]. Nonetheless, the remaining variance in these child eating behaviours is due to (non-)shared environmental influences, and parental feeding practices have been identified as one modifiable determinant.

A large number of studies have cross-sectionally examined associations between parental feeding practices and child eating behaviours such as satiety and food responsiveness, eating in the absence of hunger and self-regulation of eating [3]. These studies found mostly positive relationships between non-responsive or coercive control feeding practices (such as pressure to eat/persuasive feeding, [overt] restriction, or using food as reward/bribe) and satiety responsiveness [7, 27–32], food responsiveness [7, 27, 29, 30, 32, 33] and eating in the absence of hunger [34]. The only exception was pressure to eat which was negatively associated with food responsiveness in 3 out of 4 studies [7, 28, 30, 35]. The relationships between structure-related feeding practices and the abovementioned child eating behaviours have been less often examined. Studies found that structure-related feeding practices (i.e. family meal setting, structured meal setting, structured meal timing, established snack time) were negatively associated with satiety [7, 29] and food responsiveness [35] but positively associated with self-regulation in eating [36].

Whilst this cross-sectional research cannot determine direction of effects, recent longitudinal studies have provided support to the argument that (non-responsive) feeding practices may directly influence child eating behaviours (i.e. parent-driven association). For instance high levels of restrictive feeding practices at age 5 years were associated with more eating in the absence of hunger in girls (*n* = 140) two and 4 years later [37]; high levels of overtly restrictive feeding practices at 2 years were associated with higher satiety responsiveness at child age 3.7 years (*n* = 347) [38]; and higher levels of encouragement and emotional feeding at child age 1.5-2.5 years (*n* = 323) were prospectively associated with higher children’s tendency to overeat (combined scale including food responsiveness) 1 year later [39]. In contrast to these studies also controlling for baseline values, Gregory et al. [40] found that no association between the maternal feeding practices assessed at 2-4 years of age (*n* = 156) and food responsiveness 12 months later remained significant once the model was adjusted for baseline food responsiveness and covariates.

Although the aforementioned longitudinal studies provide some evidence to support the view that parental feeding practices can shape child eating behaviour, the extent to which parents adjust their feeding practices depending on the, at least in part genetically determined, child eating characteristics is less known (i.e. child-driven association). Rodgers and colleagues found that at child ages 1.5-2.5 years the child eating behaviour food-approach (combined scale including satiety responsiveness) was prospectively associated with less and tendency to overeat with more instrumental feeding 1 year later [39]. Whilst this study supports child-driven associations on parental feeding practices it does not assess parent-driven associations *simultaneously*.

Several studies have applied a cross-lag modelling technique recently to formally examine bidirectional effects simultaneously between parental feeding practices and child BMI [41–43], food fussiness [44], observed eating in the absence of hunger [45], and food approach/avoidance eating behaviours [46]. Steinsbekk et al. [46] examined bidirectional relationships between non-responsive feeding practices (i.e. instrumental feeding, encouragement to eat, control over eating) of 623 Norwegian parents and their child’s eating behaviours (i.e. satiety and food responsiveness along with three other behaviours) at ages 6 and 8 years. No child-driven associations were found. Instead, parental use of food as reward at 6 years predicted food responsiveness 2 years later. Despite the methodological strengths of this study, the lack of multiple assessment waves and inclusion of earlier assessment time points prevents drawing conclusions about possible prospective effects of child eating behaviours on parental feeding practices early in life.

Models of parenting and child development are typically based on a bidirectional relationship between mother and child, and it is becoming increasingly apparent that models of parent-child feeding are also likely to be reciprocal [47]. However, there is limited evidence on which to develop domain-specific accounts of the potentially bi-directional nature of parent-child feeding. Furthermore, previous longitudinal studies [37–40, 45, 46] examining the direction of relationships between parental feeding practices and child eating behaviours only considered two assessment time points with research on how children’s eating (e.g. expression of hunger and satiety cues) may influence parents’ feeding response being scant. The aim of this study was to examine bidirectional effects at child ages 2, 3.7 and 5 years between ‘authoritative’ feeding (i.e. responsive and structure-related feeding practices) and the child eating behaviours satiety responsiveness and food responsiveness. In line with the Trust model [13] that posits that responsive and structure-related feeding promotes optimal self-regulation of eating, it was predicted that non-responsive feeding would be prospectively associated with higher child food responsiveness and lower child satiety responsiveness whereas structure-related feeding would be prospectively associated with lower child food responsiveness and higher child satiety responsiveness. Additionally, it was predicted that child satiety responsiveness and food responsiveness may also predict feeding practices however due to limited theoretical and empirical research on this issue specific hypotheses were not made regarding the direction (positive or negative) of these associations.

## Methods

### Participants and procedure

This study is a secondary analysis of outcome data from the NOURISH RCT (Australian and New Zealand Clinical Trials Registry Number 12608000056392) collected at 2, 3.7 and 5 years of age and that focused on children’s healthy eating patterns and growth by promoting positive maternal feeding practices [48]. A consecutive sampling protocol was used to recruit English-speaking, first-time mothers (≥ 18 years old) delivering a healthy term baby (> 35 weeks, > 2500 g) from postnatal wards of maternity hospitals in two Australian cities between 2008 and 2009. Details of the NOURISH protocol, recruitment and participant characteristics have been described elsewhere [48, 49]. A total of 698 mothers were enrolled and then randomised at child age 4 months. Overall retention at 5 years of age was 61% [50]. Data utilised for the current study were from mothers assigned to the control only (*n* = 346) and with complete data for at least two of the three waves of data collection (*n* = 207; 60% of the control group). Data were collected via self-administered validated questionnaires at three time points: child ages 24 months (SD ± 1, range: 21-27 months), 3.7 years (SD ± 0.3, range: 3.4-4.2 years), and 5 years (SD ± 0.1, range: 4.9-5.5 years). Characteristics of the analysis sample and those excluded based on missing data are shown in Table 1. NOURISH was approved by 11 human research ethics committees including Queensland University of Technology and Flinders University.

**Table 1:** Sample characteristics assessed at child age 4 months unless otherwise specified of participants included (*n* = 207) and excluded (*n* = 139) in the current analysis

			M ± SD or count (%)	
Variable			Included	Excluded
Mother	Education level (university)*		135 (65%)	64 (46%)
	BMI		26 ± 6 (*n* = 206)	27 ± 5 (*n* = 138)
	Age at child birth (years)*		30 ± 5	29 ± 5
Child	Gender (female)*		113 (55%)	60 (43%)
	BMI-for-age z-score^a^ at age 14 months		0.46 ± 0.81	0.37 ± 0.95 (*n* = 91)

*p-value is <0.05

^a^World Health Organization standards [66]

### Maternal feeding practices

The Feeding Practices and Structure Questionnaire (FPSQ-28) assesses eight non-responsive and structure-related feeding practices that potentially influence children’s capability to self-regulate their energy intake [7, 8]. The original 40-item version has been shortened to comprise 29 items (28 items loading on 8 multi-item scales and 1 single-item-scale), scored on a 5-point Likert scale with higher scores indicating greater endorsement or more frequent use of that practice. Structure-related feeding practices included: family meal setting (1 item, “*My child eats the same meals as the rest of the family.”*), structured meal setting (3 items, e.g. “*I insist my child eats meals at the table.”, *α range = .77-.73), structured meal timing (3 items, e.g. “*I decide the times when my child eats his/her meals.”,* α range = .74-.46), and covert restriction (4 items, e.g. “*How often do you avoid buying lollies and snacks e.g., potato chips and bringing them into the house?*”*,* α range = .84-.80). Non-responsive feeding practices included: overt restriction (4 items, e.g. “*If I did not guide or regulate my child’s eating, (s)he would eat too many junk foods.*”*,* α range = .72-.55), persuasive feeding (6 items, e.g. “*When your child refuses food they usually eat, do you insist your child eats it?”,* α range = .72-.66), reward for eating (4 items, e.g. “*When your child refuses food they usually eat, do you encourage to eat by offering a food reward (e.g., dessert)?”,* α range = .84-.79), and reward for behaviour (4 items, e.g. “*I reward my child with something to eat when (s)he is well behaved.”,* α range = .81-.77). Longitudinal measurement invariance across all three time points was previously established in this study sample including the intervention group [8]. Acceptable concurrent validity, construct validity and internal reliability of the FPSQ in this study sample have also been reported [7, 8, 38]. Mean subscale scores at each time point were used in all analyses.

### Child eating behaviours

Satiety responsiveness (SR) and food responsiveness (FR) were assessed using the CEBQ [22]. Both subscales include five items such as “*My child leaves food on his/her plate at the end of a meal*” (SR, α range = .79-.72) or “*My child's always asking for food*” (FR, α range = .81-.74). Mean subscale scores for each time point were calculated with a possible range of 1 (lowest) to 5 (highest). The CEBQ has previously shown good psychometric properties (e.g. concurrent validity, internal consistency and test-retest reliability) [22, 51] and has been validated in the control group of the present sample at age 2 years (i.e. the factor structure was confirmed and all subscales showed good internal reliability with Cronbach’s alphas between 0.73 to 0.91) [52].

### Data analysis

#### Data imputation

Participants were included in the current analyses if they had provided feeding practices data at all three (*n* = 155) or at two time points (*n* = 52). Subsequent missing values of maternal feeding practices or child eating behaviour (*n* = 46) data were predicted using Expectation Maximisation (EM) imputation in SPSS Version 21.0.0 using the full dataset as well as the auxiliary variables available (e.g. child and maternal age). This ensured a complete dataset (*n* = 207) was available for the following cross-lag path models as recommended by Shin et al. [53].

#### Modelling approach

**Fig. 1:**
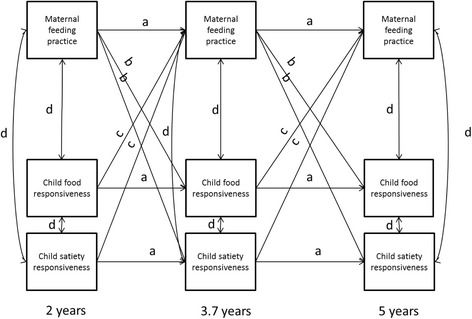
Paths estimated for each model including one of eight maternal feeding practices [8] and two child eating behaviours [22]. Models were adjusted for child BMI z-score at 14 months (not shown to enhance readability). a = autoregressive paths; b = maternal feeding practice driven paths; c = child eating behaviour driven paths; d = cross-sectional correlations

Due to the sample size the statistical power was too low to estimate a model that included all eight feeding practices along with the two child eating behaviours. Instead, one model was examined per feeding practice, including both child eating behaviours simultaneously. For each model we used path analysis (see Fig. 1) to simultaneously estimated the bidirectional paths including a) cross-lagged paths from maternal feeding practices at each time point to both child eating behaviours at the following time point and b) cross-lagged paths from both child eating behaviours at each time point to mother’s feeding practices at the following time point; along with the auto-regressive paths (continuity across time for each variable). Each model included cross-sectional correlations among the maternal feeding and child eating variables at each of the three time points. All models were adjusted for child BMI z-scores at 14 months, based on measured weight and height [48], by including regression paths from BMI z-scores to each substantive variable in the model. In the next step, non-significant paths related to BMI z-scores were trimmed for model parsimony. Maternal BMI was considered as a covariate but showed no bivariate correlations with feeding practices or child eating behaviours and was therefore not further examined. Model fit was assessed using the chi-square statistic, the root mean squared error of approximation (RMSEA), and the comparative fit index (CFI), interpreted using recommendations of Hu and Bentler [54] (RMSEA value < 0.08 and CFI value > 0.95). Following modification indices, up to two extra autoregressive paths were considered to capture associations within each variable at the 2 and 5 year time point. Analyses were conducted in Mplus Version 7.3 [55]. The standardized regression coefficients are presented.

## Results

**Fig. 2:**
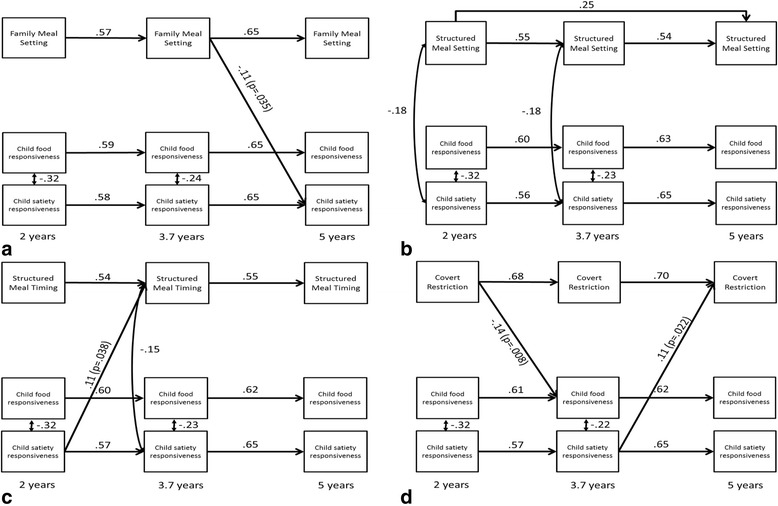
Statistically significant pathways in bidirectional models for relations among maternal-reported structure-related feeding practices: family meal setting (**a**), structured meal setting (**b**), structured meal timing (**c**), and covert restriction (**d**) and child satiety responsiveness and food responsiveness between child ages 2, 3.7 and 5 years (*N* = 207). Models were adjusted for child BMI z-score at 14 months (not shown to enhance readability). All potential cross-lagged paths from child eating behaviours to maternal feeding practices and vice versa were estimated but only statistically significant path estimates (*p* < .05) are shown here. Coefficients are standardised. Model fit across the four models was good and ranged from: *x*^2^= 27.78 to 37.41, df = 18 to 20, RMSEA = 0.05 to 0.07, CFI = 0.97 to 0.99

**Fig. 3:**
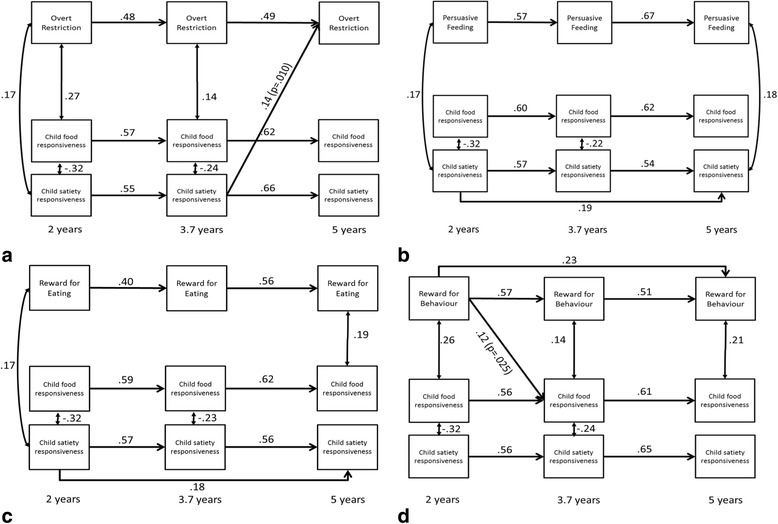
Statistically significant pathways in bidirectional models for relations among maternal-reported non-responsive feeding practices: overt restriction (**a**), persuasive feeding (**b**), reward for eating (**c**), and reward for behaviour (**d**) and child satiety responsiveness and food responsiveness between child ages 2, 3.7 and 5 years (*N* = 207). Models were adjusted for child BMI z-score at 14 months (not shown to enhance readability). All potential cross-lagged paths from child eating behaviours to maternal feeding practices and vice versa were estimated but only statistically significant path estimates (*p* < .05) are shown here. Coefficients are standardised. Model fit across the four models was good and ranged from: *x*^2^= 20.86 to 44.98, df = 17 to 20, RMSEA = 0.03 to 0.08, CFI = 0.96 to 0.99

Table 2 presents means and standard deviations of the eight maternal feeding practices, satiety responsiveness and food responsiveness. Bivariate correlations between those variables are presented in Additional file 1. The majority of maternal feeding practices and child eating behaviours increased from 2 to 3.7 to 5 years, with plateauing or decrease between the last two time points in overt restriction, covert restriction and satiety responsiveness, and decline from 2 to 3.7 to 5 years in structured meal timing. As shown in Figs. 2 and 3, there was significant continuity within the three measures of the eight feeding practices, satiety responsiveness and food responsiveness and respectively with significant autoregressive paths across time points. Child satiety responsiveness and food responsiveness were concurrently negatively correlated at the first two time points but not at the third. Structured meal setting and timing were negatively correlated with satiety responsiveness at the first two time points and the second time point respectively, while neither was at the third time point. The four non-responsive feeding practices showed positive correlations with satiety responsiveness and food responsiveness at various time points (see Fig. 3).

**Table 2:** Means and standard deviations of the eight maternal feeding practices^a^, satiety responsiveness and food responsiveness^b^ at 2, 3.7 and 5 years of age (*n* = 207)

	2 years	3.7 years	5 years
Family meal setting	3.71 ± 1.27	4.07 ± 1.16	4.39 ± 0.96
Structured meal setting	3.96 ± 0.72	4.11 ± 0.66	4.31 ± 0.59
Structured meal timing	3.88 ± 0.59	3.80 ± 0.52	3.76 ± 0.52
Covert restriction	3.20 ± 0.90	3.28 ± 0.80	3.24 ± 0.78
Overt restriction	3.40 ± 0.83	3.54 ± 0.91	3.46 ± 0.84
Persuasive feeding	2.70 ± 0.60	3.12 ± 0.62	3.14 ± 0.60
Reward for eating	1.81 ± 0.71	2.54 ± 0.73	2.58 ± 0.71
Reward for behaviour	1.85 ± 0.69	2.20 ± 0.74	2.22 ± 0.73
Satiety responsiveness	3.00 ± 0.59	3.00 ± 0.57	2.98 ± 0.56
Food responsiveness	2.27 ± 0.69	2.41 ± 0.66	2.44 ± 0.65

^a^Maternal feeding practices were measured with the Feeding Practices and Structure Questionnaire [8]; possible response range is 1-5 with higher scores indicating more endorsement of the practice

^b^Satiety responsiveness and food responsiveness were measured with the Child Eating Behaviour Questionnaire [22]; possible response range is 1-5 with higher scores indicating more endorsement of the behaviour

Out of the 64 cross-lag paths across the eight models, six were found to be significant. No significant cross-lag paths were found in the models for structured meal setting, persuasive feeding and reward for eating. Significant cross-lags paths in the other models suggest a mix of mother-driven and child-drive associations. In terms of mother-driven associations, three feeding practices were associated with subsequent child eating behaviour. Lower scores on covert restriction and higher scores on reward for behaviour at 2 years were associated with higher food responsiveness at 3.7 years. A lower score on family meal setting at 3.7 years was associated with higher scores on satiety responsiveness at 5 years. In terms of child-driven associations, satiety responsiveness was prospectively associated with three subsequent maternal feeding practices. Higher satiety responsiveness at 2 years was associated with higher scores on the structured meal timing variable at 3.7 years. Satiety responsiveness at 3.7 years was positively associated with covert and overt restriction at 5 years.

## Discussion

This study is the first to use cross-lag modelling to examine bidirectional relationships between maternal feeding practices and two child eating behaviours of satiety responsiveness and food responsiveness across three time points in the pre-school years. These maternal practices and child eating behaviours have been hypothesised to be associated with child obesity risk [3, 20]. Relationships between variables were tested across three time points spanning from child age 2-5 years with the same method used to assess variables at each time point. All models provided strong and consistent evidence for longitudinal stability of all maternal feeding practices and both child eating behaviours with 16-49% of variance explained by the practice or behaviour at the previous years (vs 18-53% of variance explained by all previous and concurrent variables). A small number of both mother-driven and child-driven independent associations were found after adjusting for child BMI z-score at 14 months and all relevant autoregressive and cross-sectional associations. Two maternal feeding practices (reward for behaviour and covert restriction) were prospectively positively and negatively associated with food responsiveness from 2 to 3.7 years of age, with a third practice (family meal setting) at 3.7 years inversely associated with satiety responsiveness at 5 years of age. Three child-driven associations were seen for satiety responsiveness but none for food responsiveness. Satiety responsiveness at 2 years was prospectively positively associated with structured meal timing at 3.7 years of age and with both covert and overt restriction from 3.5-5 years.

Overall the findings provided limited support for the theoretical perspective that the provision of structure and responsiveness to a child’s cues of hunger and satiety fosters the ability to self-regulate energy intake [11–13], which in this study is operationalised as food responsiveness and satiety responsiveness [22]. In line with predictions, there was some support for the notion that non-responsive and structure-related feeding practices could influence the expression of food responsiveness over time. Specifically our current findings showed that less frequent use of covert restriction (i.e. structure-related feeding practice) and more frequent use of reward for behaviour (i.e. non-responsive feeding practice) at 2 years of age, predicted higher food responsiveness at 3.7 years; although there was no comparable effect between 3.7 and 5 years. There were no effects of the other non-responsive feeding practices (persuasive feeding and reward for eating) on child eating behaviours at any age. Previous longitudinal research has shown parent-driven associations for food responsiveness. Steinsbekk et al. [46], conducting cross-lag analysis across two time points in older children (6-8 years), found that instrumental feeding, which is similar to reward for behaviour as used in our study, predicted more food responsiveness 2 years later. Similarly, in a sample that overlaps in age to that in our study, Rodgers et al. [39] found that encouragement/prompting assessed at child age 1.5-2.5 years positively predicted the child’s tendency to overeat (external eating, desire to drink and food responsiveness) 1 year later. In addition, maternal emotional feeding (emotional feeding and food to calm) also prospectively predicted higher tendency to overeat at around 3 years of age. Notably, these relationships were not adjusted for child BMI z-scores.

The current analysis does not provide any evidence for the predicted relationships between feeding practices and satiety responsiveness from 2 to 5 years of age. While one association was significant – family meal setting at 3.7 years was prospectively negatively associated with satiety responsiveness at 5 years of age – the direction of the effect was contrary to the hypothesis. The finding was also contrary to a previous cross-sectional study that found a positive association between family meal setting and self-regulation in eating (assessed with the self-regulation in eating scale by Tan and Holub [56]), but only in overweight pre-schoolers [36]. Although the satiety responsiveness scale of the CEBQ was designed to indicate the extent to which a child responds to intrinsic cues of hunger and satiety [18], it has been suggested that mothers may interpret their child’s satiety responsiveness (small appetite and not eating enough) as a cause for concern and see their child as a problem eater [7]. How mothers in the present sample interpreted the items on this scale (e.g. fills up easily, leaves food on the plate) is beyond the scope of the present study. However, the potential issue with this scale in the present study is also highlighted in some of the child-driven associations that were found.

Child-driven associations were limited to satiety responsiveness which predicted more structure (i.e. mother decides timing of snacks and meals) at 3.7 years and more restriction, both overt and covert, at 5 years. These findings suggest that a mother might adjust her feeding practices in response to her perception of the child’s satiety responsiveness. In line with the afore mentioned speculation that mothers may perceive behaviours reflecting higher satiety responsiveness as an indicator that their child is a ‘bad eater’ [7], satiety responsiveness in the child may elicit more frequent structure related to meal timing and restriction (both covert and overt) as a way of preventing the child with a small appetite from ‘filling up’ on unhealthy foods. Clearly further research is needed to better understand mothers’ responses to smaller child appetite.

The consistency and strength of the autoregressive associations of both feeding practices and child eating behaviours across the three time points are notable. This is in line with previous research showing stability within feeding practices [26, 40, 57, 58] and child eating behaviours [25, 26]. The longitudinal stability of the eating behaviours is also in line with the evidence of the heritability of these traits. Nevertheless, well over half the variance in the practices/behaviours at each age is not explained by the temporal associations and is lower than the proposed heritability estimates of the latter (> 70%) [23, 24]. The autoregressive effect sizes were larger than the modest cross-lag associations between practices and behaviours. One interpretation is that both maternal feeding practices and child eating behaviour are established by 2 years of age and that there is only limited scope beyond infancy for interventions that target maternal feeding practices to modify child eating behaviour as a mediator of child obesity risk. This is consistent with child development models and the life course approach both of which suggest that very early interventions have potential to take advantage of both biological and behavioural plasticity [50, 59]. The data presented here provide no evidence on potential direction and strength of bidirectional relationships prior to 2 years of age.

The present analyses highlight the variable, complex bidirectional relationships between maternal feeding practices and child eating behaviours. The six significant cross-lag pathways provide limited evidence that child satiety responsiveness elicits a maternal feeding response (meal/snack timing and restriction) whereas mother-driven associations (reward and restriction) are mostly limited to child food responsiveness. There were 13 significant cross-sectional associations between feeding practices and eating behaviours, ten of which were between non-responsive practices and child eating behaviour. Given the strength and consistency of the temporal associations we have reported in both sets of variables, these multiple cross-sectional associations at various ages that have also been frequently reported in the literature may reflect relationships between feeding practices and child eating behaviour established at a younger age, rather than at the specific age where the cross-sectional associations are demonstrated. It is notable that there appeared to be no cross-lag effects in either direction for persuasive feeding or reward for eating, both of which have been the focus of many cross-sectional studies and are widely seen as coercive controlling feeding practices associated with obesity risk [3, 17, 60]. Pressure to eat has also previously been positively associated with satiety responsiveness (mostly cross-sectional) and eating in the absence of hunger (one cross-lag study [45] and one experimental study [34]). Differences in these studies may be due to the study design, as well as the sample size and measures of the feeding practice (persuasive feeding vs. pressure to eat) and child eating behaviour (satiety responsiveness vs. kcal consumed in the absence of hunger). It must be acknowledged that the significant cross-lag paths are relatively few and the effect sizes modest. However, the sample size is relatively small and a further eight pathways (four mother- and four child-driven) returned a *p*-value > 0.05-0.1 (data not shown); hence the possibility of Type II error arises.

One plausible interpretation of these results is that when comprehensive modelling strategies such as the cross-lagged analyses that are presented here are used, parental feeding practices (as currently measured) have little influence on what are now widely agreed to be heritable child eating behaviours (as currently measured) and hence, call into question the widely articulated rationale for early feeding interventions. However, high quality evidence from the NOURISH RCT [48] indicates that modifying maternal feeding practices results in small but significant changes in child eating behaviour, particularly satiety and food responsiveness up to 5 years of age [61]. Although these intervention effects appeared modest, these results suggest that, as with many health behaviour phenotype [62], there are both genetic and environmental influences on eating behaviour. The NOURISH intervention was designed in 2008, prior to publication of the GEMINI data that provided evidence of a strong genetic component to child eating behaviours [19] and coined the term ‘appetitive traits’ [20]. As such, the intervention was universal and delivered independent of obesity risk or obesogenic child appetitive traits. A further critical consideration is that the NOURISH intervention was delivered prior to 18 months of child age in contrast to the current analysis that is confined to 2-5 years of age. A more targeted intervention that provided mothers with feeding advice tailored to their child’s individual appetite ‘profile’ may result in modification of the child’s appetite phenotype and potentially reduce obesogenic eating behaviour and risk.

Findings of this study have to be considered in light of the strengths and limitations of the design. Strengths of the study include utilising three assessment time points in early childhood at which the same measurement tools were utilise to assess a range of maternal feeding practices and two child eating behaviours. All models examining the relationships between maternal feeding practices and child eating behaviours were adjusted for child BMI z-score. Child actual weight or perceived weight status has been shown to be associated with both maternal feeding practices and the appetite traits considered here [3, 17, 20, 60]. While two of the feeding practices showed internal reliability values below the desirable cut-off and findings need to be considered in light of this potential limitation, the current study included non-responsive and structure-related feeding practices, thus allowing for an analysis of comprehensive influences [42, 63] on children’s eating behaviours and vice-versa. While the size of effects presented here were small, these are in line with previously reported standardised estimates of ≤ 0.2 [39–43] and are in particular comparable to Steinbekk et al.’s cross-lag model findings [46] based on a much larger sample size and across just two age points. Notably, these modest associations are independent of previous levels of both feeding practices and child eating behaviours which appear to be highly stable and therefore possibly set early in life [46, 64]. Due to the small sample size it was not feasible to test all maternal feeding practices variables simultaneously as done by Steinsbekk and colleagues [46]. Data for this secondary data analysis were collected via the same mother-reported validated questionnaires at each time point. Questionnaire-based assessment of both feeding practices and child eating behaviour are widely used, particularly for larger samples. The same approach was used in two previously published papers examining cross-lagged relationships between feeding practices and child eating behaviours [44, 46]. However it is possible that using self-report for both measures may result in common method variance across measures and time points and thus the same reporting bias. Furthermore, as for all self-report measures, there is potential for social desirability bias. It should be noted that the alternate approach of direct observation is also subject to measurement limitations including feasibility for large samples, one-off observation and quasi-experimental setting of the observations that may result in behaviour change of both parent and child, and observer/coding bias and reliability [65]. Nonetheless, future studies should replicate these analyses with observational data.

## Conclusion

The present study is the first to utilise a cross-lagged analysis to investigate bidirectional relationships between maternal feeding practices and child eating behaviours over three ages.

A key finding was the high degree of consistency of both feeding practices and child eating behaviours across the pre-school years with evidence of both maternal- and child-driven associations. Overall the results suggest fewer maternal-driven associations with child eating behaviours than have been reported in previous comparatively unadjusted cross-sectional and longitudinal studies. Nevertheless, there is some evidence that coercive/non-responsive feeding practices are associated with small increases in child food responsiveness. These results suggest the need for targeted interventions, at least those across the preschool years, which are tailored to assist mothers to understand and respond appropriately to their child’s individual appetite profile.

## Additional file

Additional file 1: Bivariate correlations between child eating behaviours and maternal feeding practices (*N* = 207). (DOCX 21 kb)

## Abbreviations

BMI: Body mass index
CEBQ: Children’s eating behaviour questionnaire
CFI: Comparative fit index
CFQ: Child feeding questionnaire
FPSQ: Feeding practices and structure questionnaire
FR: Food responsiveness
RMSEA: Root mean squared error of approximation
SR: Satiety responsiveness
