# Supplementary material for: Bidirectional associations between mothers’ feeding practices and child eating behaviours
Source: Int J Behav Nutr Phys Act. 2018 Jan 11;15:3. doi: 10.1186/s12966-018-0644-x (PMC5765660; doi:10.1186/s12966-018-0644-x)
Supplement: Additional file 1: — Bivariate correlations between child eating behaviours and maternal feeding practices (N = 207). (DOCX 21 kb) [file 12966_2018_644_MOESM1_ESM.docx]

Additional file 1: Bivariate correlations between child eating behaviours and maternal feeding practices (N = 207)

|  | SR | | FR | | | RB | | | RE | | | PF | | | OR | | | CR | | | FMS | | | SMT | | | SMS | | |
| --- | --- | --- | --- | --- | --- | --- | --- | --- | --- | --- | --- | --- | --- | --- | --- | --- | --- | --- | --- | --- | --- | --- | --- | --- | --- | --- | --- | --- | --- |
|  | SR2 | SR3 | FR1 | FR2 | FR3 | RB1 | RB2 | RB3 | RE1 | RE2 | RE3 | PF1 | PF2 | PF3 | OR1 | OR2 | OR3 | CR1 | CR2 | CR3 | FMS1 | FMS2 | FMS3 | SMT1 | SMT2 | SMT3 | SMS1 | SMS2 | SMS3 |
| SR1 | .63** | .54** | -.32** | -.29** | -.18* | .09 | .03 | -.06 | .18* | .12 | .08 | .18* | .16* | .10 | .17* | .12 | .14* | -.03 | -.04 | -.02 | -.10 | -.21** | -.16* | -.00 | .10 | -.05 | -.19** | -.03 | -.10 |
| SR2 |  | .72** | -.14 | -.28** | -.18* | .11 | .13 | .10 | .07 | .15* | .15* | .07 | .13 | .16* | .20** | .20** | .23** | .05 | -.02 | .10 | .05 | -.03 | -.10 | -.01 | -.05 | -.07 | -.14 | -.16* | -.21** |
| SR3 |  |  | -.21** | -.31** | -.18* | .03 | .07 | .06 | .01 | .04 | .13 | .07 | .08 | .18* | .12 | .07 | .16* | -.09 | -.11 | -.00 | -.08 | -.13 | -.17* | -.02 | -.06 | -.02 | -.11 | -.13 | -.18* |
| FR1 |  |  |  | .63** | .54** | .28** | .26** | .23** | .11 | .09 | .09 | -.02 | .05 | .02 | .27** | .18* | .17* | .09 | .09 | .07 | .05 | .07 | .09 | -.03 | -.03 | -.08 | -.02 | -.06 | -.09 |
| FR2 |  |  |  |  | .70** | .28** | .31** | .28** | .10 | .11 | .14 | .05 | .02 | .02 | .23** | .25** | .15* | -.09 | .00 | .00 | .07 | .06 | .10 | .01 | .02 | .02 | .05 | .05 | .04 |
| FR3 |  |  |  |  |  | .23** | .26** | .34** | .05 | .09 | .23** | .07 | .02 | .06 | .11 | .25** | .20** | -.01 | -.00 | .00 | -.01 | .01 | .00 | .05 | .07 | -.00 | -.05 | -.03 | -.06 |
| RB1 |  |  |  |  |  |  | .60** | .56** | .54** | .33** | .32** | .36** | .26** | .26** | .22** | .16* | .13 | -.06 | -.04 | .05 | -.10 | -.11 | -.08 | -.15* | -.05 | -.12 | -.25** | -.17* | -.19** |
| RB2 |  |  |  |  |  |  |  | .67** | .27** | .36** | .37** | .25** | .25** | .26** | .08 | .23** | .20** | -.01 | .02 | .03 | .01 | -.05 | -.02 | -.14* | -.14* | -.21** | -.18** | -.11 | -.13 |
| RB3 |  |  |  |  |  |  |  |  | .21** | .25** | .51** | .25** | .23** | .35** | .09 | .16* | .24** | -.01 | -.02 | .04 | -.08 | -.01 | -.05 | -.01 | -.08 | -.13 | -.11 | -.14* | -.11 |
| RE1 |  |  |  |  |  |  |  |  |  | .42** | .19** | .50** | .31** | .24** | .14* | .12 | .07 | -.03 | .01 | .07 | -.09 | -.13 | -.03 | .00 | .07 | .03 | -.18** | -.01 | .022 |
| RE2 |  |  |  |  |  |  |  |  |  |  | .57** | .31** | .34** | .28** | .15* | .18* | .12 | .09 | .05 | .07 | -.20** | -.26** | -.14* | -.00 | -.01 | -.02 | -.16* | -.09 | -.07 |
| RE3 |  |  |  |  |  |  |  |  |  |  |  | .29** | .26** | .45** | .07 | .15* | .15* | .04 | -.02 | -.01 | -.24** | -.23** | -.28** | .11 | -.03 | -.06 | -.10 | -.12 | -.05 |
| PF1 |  |  |  |  |  |  |  |  |  |  |  |  | .58** | .49** | .11 | .01 | .09 | -.07 | -.12 | -.00 | -.16* | -.15* | -.08 | .22** | .12 | .07 | -.12 | -.07 | -.05 |
| PF2 |  |  |  |  |  |  |  |  |  |  |  |  |  | .68** | .20** | .14* | .12 | .02 | -.04 | .07 | -.09 | -.15* | -.01 | .24** | .17* | .12 | -.09 | .06 | .07 |
| PF3 |  |  |  |  |  |  |  |  |  |  |  |  |  |  | .09 | .11 | .13 | -.08 | -.16* | -.01 | -.04 | -.08 | -.11 | .06 | -.03 | .02 | -.05 | -.01 | .08 |
| OR1 |  |  |  |  |  |  |  |  |  |  |  |  |  |  |  | .50** | .39** | .18* | .10 | .13 | .05 | -.03 | .01 | .11 | .08 | .09 | -.09 | -.08 | -.04 |
| OR2 |  |  |  |  |  |  |  |  |  |  |  |  |  |  |  |  | .54** | .20** | .22** | .22** | -.01 | -.10 | -.04 | .08 | .10 | -.08 | -.16* | -.14* | -.12 |
| OR3 |  |  |  |  |  |  |  |  |  |  |  |  |  |  |  |  |  | .20** | .20** | .28** | -.03 | -.06 | -.06 | .02 | -.03 | -.020 | -.17* | -.19** | -.17* |
| CR1 |  |  |  |  |  |  |  |  |  |  |  |  |  |  |  |  |  |  | .69** | .58** | -.01 | .01 | .10 | .11 | .12 | .09 | .13 | .06 | .07 |
| CR2 |  |  |  |  |  |  |  |  |  |  |  |  |  |  |  |  |  |  |  | .70** | -.05 | .04 | .10 | .08 | .12 | .02 | .06 | .03 | .03 |
| CR3 |  |  |  |  |  |  |  |  |  |  |  |  |  |  |  |  |  |  |  |  | -.02 | -.02 | .06 | .11 | .07 | -.01 | .05 | -.08 | -.02 |
| FMS1 |  |  |  |  |  |  |  |  |  |  |  |  |  |  |  |  |  |  |  |  |  | .58** | .48** | -.04 | .05 | .07 | .13 | .16* | .15* |
| FMS2 |  |  |  |  |  |  |  |  |  |  |  |  |  |  |  |  |  |  |  |  |  |  | .65** | -.04 | .09 | .15* | .19** | .15* | .21** |
| FMS3 |  |  |  |  |  |  |  |  |  |  |  |  |  |  |  |  |  |  |  |  |  |  |  | .06 | .24** | .26** | .20** | .17* | .26** |
| SMT1 |  |  |  |  |  |  |  |  |  |  |  |  |  |  |  |  |  |  |  |  |  |  |  |  | .54** | .45** | .15* | .12 | .07 |
| SMT2 |  |  |  |  |  |  |  |  |  |  |  |  |  |  |  |  |  |  |  |  |  |  |  |  |  | .55** | .22** | .27** | .21** |
| SMT3 |  |  |  |  |  |  |  |  |  |  |  |  |  |  |  |  |  |  |  |  |  |  |  |  |  |  | .18** | .16* | .29** |
| SMS1 |  |  |  |  |  |  |  |  |  |  |  |  |  |  |  |  |  |  |  |  |  |  |  |  |  |  |  | .54** | .56** |
| SMS2 |  |  |  |  |  |  |  |  |  |  |  |  |  |  |  |  |  |  |  |  |  |  |  |  |  |  |  |  | .69** |

Abbreviations: SR = Satiety Responsiveness, FR = Food Responsiveness, RB = Reward for Behaviour, RE = Reward for Eating, PF = Persuasive Feeding, OR = Overt Restriction, CR = Covert Restriction, FMS = Family Meal Setting, SMT = Structured Meal Timing, SMS = Structured Meal Setting
